# Pregnancy and Asymptomatic Carriage of *Pneumocystis jiroveci*

**DOI:** 10.3201/eid0905.020660

**Published:** 2003-05

**Authors:** Sergio L. Vargas, Carolina Angelica Ponce, Catherine Andrea Sanchez, Ana Victoria Ulloa, Rebeca Bustamante, Guido Juarez

**Affiliations:** * University of Chile School of Medicine, Santiago, Chile; † University of Chile Hospital, Santiago, Chile

**To the Editor:** Severe immunosuppression is the leading determinant host factor for *Pneumocystis* pneumonia (PCP) (*1*). However, PCP is not restricted to those who are severely immunocompromised. Molecular techniques based on the amplification of specific regions of *P. jiroveci* (human-derived *Pneumocystis*) DNA by using polymerase chain reaction (PCR) in noninvasive human samples suggest that the infection is common in other segments of the population that are immunocompetent or display a lesser degree of immune compromise (*2*,*3*). A mild or asymptomatic form of *P. jiroveci* infection, or a carrier state, likely develops in these persons, who may play a role in the circulation of *P. jiroveci* in the community while serving as silent reservoirs for transmission to susceptible persons. This description fits infants who acquire the primary *Pneumocystis* infection very early in life, patients with chronic respiratory disorders, elderly adults, and other groups (*2*,*3*). Extensive searches have been unsuccessful in detecting carriage of *P. jiroveci* DNA in noninvasive samples (i.e., nasal and throat swabs, saliva) from immunocompetent healthy adults (*4*).

Evidence suggests that latency of *P. jiroveci* is time-limited and that PCP is more likely an actively acquired infection (*1*). Characterization of potentially infectious reservoirs might lead to new intervention strategies to prevent transmission. Furthermore, the detection of *P. jiroveci* strains with mutations at the dihydropteroate synthase locus, which in other pathogens confer resistance to trimethoprim-sulfamethoxazole, suggests that resistance to this primary therapy of PCP may be emerging (*1*). New strategies for *P. jiroveci* prophylaxis may soon be needed.

Evidence suggests that normal pregnancy may be accompanied by changes in the immune response that may in part account for the successful growth and delivery of the “fetus hemi-allograft.” A subtle shift from the response of Th1 (cellular immunity) CD4+ lymphocytes to a proportional increase in the Th2 (humoral immunity) CD4+ response can be detected (*5*). These responses have not been clearly explained but would most likely occur because of shifts in the production of cytokines, impairing defense against certain infections. Pregnancy’s important hormonal changes (e.g., increases in the secretion of human chorionic gonadotropin, progesterone, estrogen, corticosteroids, α-fetoprotein, prolactin, and α-globulin) may also contribute to decreased resistance. While overt immune deficiency is difficult to detect, an increase in some viral infections has been documented, which may indicate a gentle form of depressed immune response (*6*). In addition, this physiologic compensation generates an increase in illness and death from other infections that require a protective Th1 response as, for example, tuberculosis, malaria, American trypanosomiasis, leishmaniasis, toxoplasmosis, lysteriosis, and pneumocystosis. Reports indicate that illness in HIV-infected women with PCP is greater when the women are pregnant (*7*). However, no data show that pregnant women may be asymptomatic carriers of *P. jiroveci*.

A prospective, pilot study of 33 third-trimester, pregnant, asymptomatic healthy women and 28 healthy women within 15 days of a menstrual period (controls) was conducted. Participants were followed at an outpatient clinic in Santiago during January through March 2002. Ages were 14–39 years (median 26 years) for pregnant women and 17–45 years (median 28 years) for controls. Previous pregnancies ranged from 0 (n=10) to 4 (median 1) for pregnant women and from 0 (n=9) to 3 (median 1) for controls. *P. jiroveci* was detected in deep nasal swab samples in a nested-PCR procedure by using oligonucleotide primers pAZ102E and pAZ102H. (These primers were designed for the gene encoding the mitochondrial large subunit rRNA of rat-derived *Pneumocystis* [*P. carinii*] that amplifies all forms of *Pneumocystis* and internal primers pAZ102X and pAZ102Y, specific for *P. jiroveci*.) DNA extraction was performed with a commercial kit (QIAamp DNA mini kit; Qiagen Inc., Valencia, CA). Positive, negative, and internal control primers, directed to the human globin gene to detect sample inhibition and verify successful extraction, were used during the DNA amplification procedure. Samples were processed under a laminar flow hood to prevent contamination, and PCR assays were repeated twice. The Ethics Committee of the University of Chile School of Medicine approved the study.

Five (15.5%) of the 33 pregnant women had *P. jiroveci* DNA in their nasal swab samples versus none (0%) of the 28 nonpregnant controls (p=0.04 by 1-sided Fisher exact test). Immunologic parameters were not tested. The *P. jiroveci*–positive women were all multiparous with 1 (n=2), 2 (n=2), or 3 (n=1) previous pregnancies.

These results suggest that pregnancy is a host factor that favors asymptomatic nasal carriage of *P. jirovec*. However, PCR detection of *P. jiroveci* DNA in the nares of pregnant women does not necessarily indicate either a mild active pulmonary infection or viable or transmissible organisms. In animal models, detection of *P. carinii* DNA in nasal and oral samples is a good indicator that *Pneumocystis* is in the lungs (*8*).

These results also support the hypothesis that pregnant women who nasally carry *P. jiroveci* may play a role as contagious sources for susceptible persons, especially their immunologically naive newborn infants. This hypothesis warrants further study. Mother-to-infant transmission may explain the accumulating evidence that the primary infection is widely acquired very early in life (*9*). Recent animal model studies have documented the early acquisition of *P. carinii* (within 1 to 2 h after birth) in neonatal rats, likely transmitted by the dams (*10*). Evidence of mother-offspring transmission would be clinically relevant for infants born to HIV-infected mothers, who currently rely on empiric anti-*Pneumocystis* chemotherapy started at 1 month of age as their only prophylactic option.
